# Mechanical study of the application of compression screw nails in the cross-inverted triangular pattern for internal fixation of femoral neck fractures

**DOI:** 10.1186/s12891-023-06297-x

**Published:** 2023-05-05

**Authors:** Min Wang, Yunlong Wang, Fa Zou, Lin Tan, Yunjuan Wang

**Affiliations:** 1 Orthopedics, Chengdu Yumei Hospital, Sichuan, China; 2 School of Basic Medicine, Chongqing Medical University, Chongqing, China; 3 Department of Oncology , Sichuan Provincial People’s Hospital, University of Electronic Science and Technology of China, Chengdu, China; 4 Department of Radiology Oncology, West China Hospital of Sichuan University, Chengdu, China

**Keywords:** Cross-inverted triangular pattern, Fracture fixation, Femoral neck fracture, Biomechanics

## Abstract

**Objective:**

To design a cross-inverted triangular pattern to insert compression screw nails for the treatment of femoral neck fractures and to compare the biomechanics of inserting compression screw nails in cross-inverted triangular patterns and inverted triangular patterns.

**Methods:**

The reasonableness of the model is first analyzed using finite elements. A total of 6 adult human specimens were selected, of which 3 males and 3 females were divided into the A1, B1, and C1 groups and the A2, B2, and C2 groups by the random number table method. The A1 and A2 groups were made into subhead femoral neck fracture models, the B1 and B2 groups were made into trans-neck femoral neck fracture models, and the C1 and C2 groups were made into basal femoral neck fracture models. The right femur of each group had a compression screw nail inserted in the crossed-inverted triangular pattern, and the left femur of each group had a compression screw nail inserted in the inverted triangular pattern. The static compression test was performed by an electronic universal testing machine. The maximum load of the femoral neck and the load of 3.00 mm axial displacement of the femoral head were read according to the pressure-displacement curve drawn in the experiment.

**Results:**

The finite element analysis showed that the cross-inverted triangular hollow threaded nail has better conductivity and more stable fixation than the inverted triangular hollow threaded nail. The maximum load of the femoral neck and the load of 3.00 mm axial displacement of the femoral head of the left femur were greater than those of the right femur in the A1, A2, B1, B2 and C2 groups, while the maximum load of the femoral neck and the load of 3.00 mm axial displacement of the femoral head of the left femur were smaller than those of the right femur in the C1 group. There was no statistically significant difference in the maximum load of the femoral neck or the load of 3.00 mm axial displacement of the femoral head between the A1 and A2 groups, the B1 and B2 groups, or the C1 and C2 groups (P > 0.05). After the K-S test, the maximum load of the femoral neck and the load of 3.00 mm axial displacement of the femoral head were normally distributed (P = 0.20), and the LSD-t test was conducted for the two load data; the difference was not statistically significant (P = 0.235).

**Conclusion:**

The effect of compression screw nails in the cross-inverted triangular pattern was the same in males and females, and stability was better in the fixation of subhead and trans-neck femoral neck fractures. However, its stability in fixation of basal femoral neck fracture is worse than that of the inverted triangular pattern. The cross-inverted triangular hollow threaded nail has better conductivity and more stable fixation than the inverted triangular hollow threaded nail.

## Highlight

Proposing a new internal fixation modality to treat femoral neck fractures.

Use finite element analysis and Biomechanical analysis together to verify the rationality of the theory.

## Background

Femoral neck fracture is a common fracture of the hip, and the incidence of femoral neck fracture in young adults at home and abroad has been increasing year by year in recent [1, 2]. Treatment of femoral neck fracture by internal fixation can achieve anatomical repositioning, restore or maintain stable blood supply to the femoral head, and prevent complications such as ischemic necrosis and poor fracture [3]. Currently, compression threaded nails are preferred in the treatment of femoral neck fractures, and most scholars believe that three compression threaded nails are more stable than two. According to the literature, inadequate placement and fixation strength of the compression nail can cause loosening and displacement due to low compressive stress, resulting in poor fracture [4–9]. Currently, the inverted triangular approach is commonly used to place compression nails, which means that the lower nail is positioned in the middle of the femoral spur, and then two nails are placed parallel to each other above the middle of the femoral head, forming an inverted “zigzag” [10]. However, due to the special anatomical characteristics of the femoral neck, the risk of fracture nonunion and femoral head necrosis is higher. Previously, femoral neck shortening was clinically considered to be a common phenomenon after internal fixation of femoral neck fractures, and existing studies have shown that different internal fixation methods have different degrees of influence on femoral neck [11–13]. To reduce the occurrence of femoral neck shortening, our group designed a cross-inverted triangle nailing method: based on the inverted triangle method, the upper two pressurized nails were driven into the femur at the same entry point at a certain angle, while the third pressurized nail remained in the same position, forming a cross-inverted triangle to increase the contact area between the nail and the femoral medulla. To determine the superiority of this nailing method, the biomechanical stability of the cross-inverted triangle nailing method was compared with that of the inverted triangle nailing method by using finite element analysis, and its biomechanical test parameters on femoral neck fractures were compared with those of the inverted triangle nailing method, which are reported as follows.

## Finite element analysis

### Time and location

The experiments were completed at the Biomechanics Research Center of Chongqing Medical University from January 2022 to March 2022.

### Materials

One adult healthy male volunteer, age 50 years, height 170 cm, body mass 70 kg, was selected to obtain his raw CT data of the proximal femur. We used femur CT data for modeling to perform finite element analysis.The volunteer gave informed consent to the experiment and signed the informed consent form.

The original DICOM data were acquired by a Light Speed 16-row spiral CT manufactured by General Electric (GE) with the following scanning conditions: scanning current of 100 mA, scanning voltage of 120 kV, bone tissue window scanning, layer interval of 0.75 mm, layer thickness of 1.5 mm; 3D reconstruction software provided by Magisterial, Belgium MIMICS 21.0 software from Magisterial, Belgium; appropriate grayscale values were selected to differentiate the tissue from the bone, mask editing and area growth were applied to create a 3D model of the femur, and the model file was exported in STL format. Geo-magic Wrap 2017 software was used to remove features, reduce noise, perform mesh division, fit surfaces, etc. After completing the optimization of the original model, the resulting 3D model of callousness and cortical bone of the femur was saved in STEP format. Geomagic Wrap 2017 software was used to remove features, reduce noise, mesh, and fit surfaces to the images, and after the original model optimization, the resulting 3D models of callousness bone and cortical bone were saved in STEP format.

### Model establishment

**Fig. 1:**
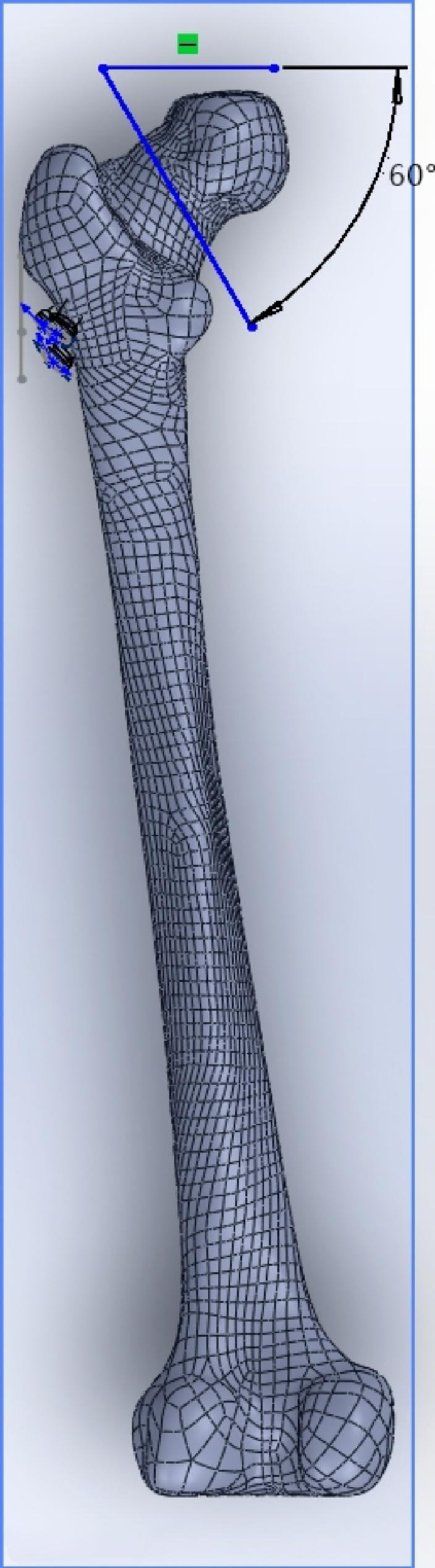
Transcervical femoral neck fracture model

The femoral model was processed using Solid-works 2017 software to create a cross section at the center of the femoral head to create a cutting plane, ensuring that the cutting plane was located near the center of the femoral neck and at a 30° angle to the cross section to complete the transcervical fracture of the femoral neck (Fig. 1). Two internal fixation models were created using the same software, with a hollow screw diameter of 7.3 mm and a thread length of 16 mm. In both internal fixation models, three hollow screws were placed in the form of cross-inverted triangles and inverted triangles. The complete models were imported into Abacus 2017 software, and material property assignment, boundary condition constraint, load distribution and meshing operations were performed in turn; each assembly was meshed with tetrahedral cells.

### Test method and indicators

To simulate the human standing condition and ensure the load force vector in the coronal plane with the femoral stem axis and digitalis plane angle of 13° and 8°, respectively, the relationship between the internal fixation device and the femur was chosen to bind contact, and the friction coefficient of the fracture surface was set to 0.46. The stress distribution and the maximum equivalent force of the femur and the internal fixation in the two groups of models were analyzed.

## Biomechanical analysis

### Experimental materials and instruments

Six adult body specimens, three of each sex, were selected from the anatomy department of Chongqing Medical University. They were examined before osteotomy, and no deformity in appearance and no bone disease were found. A 7.30 mm diameter pressurized threaded nail. c43.104 electronic universal testing machine was purchased from Meters Industrial Systems Ltd. The anatomical experiments of this study were completed on April 5, 2019, at the anatomy teaching and research laboratory of Chongqing Medical University, and the biomechanical tests were completed on July 12, 2019, at Meingi Medical Technology Co.

### Methods

Rock-wood fracture models were [12]: three male specimens were divided into groups A1, B1, and C1, and three female specimens were divided into groups A2, B2, and C2 by the random number table method. The femoral specimens were cut off with a chainsaw: the Al and A2 groups were made into subcranial femoral neck fracture models, the B1 and B2 groups were made into trans-cervical femoral neck fracture models, and the C1 and C2 groups were made into basal femoral neck fracture models. In each group, the left branch was nailed in an inverted triangular fashion, and the right branch was nailed in a cross-inverted triangular fashion. To ensure the freshness of the femoral specimens, the fracture models were placed in the freezer and removed during the mechanical experiments.

**Fig. 2:**
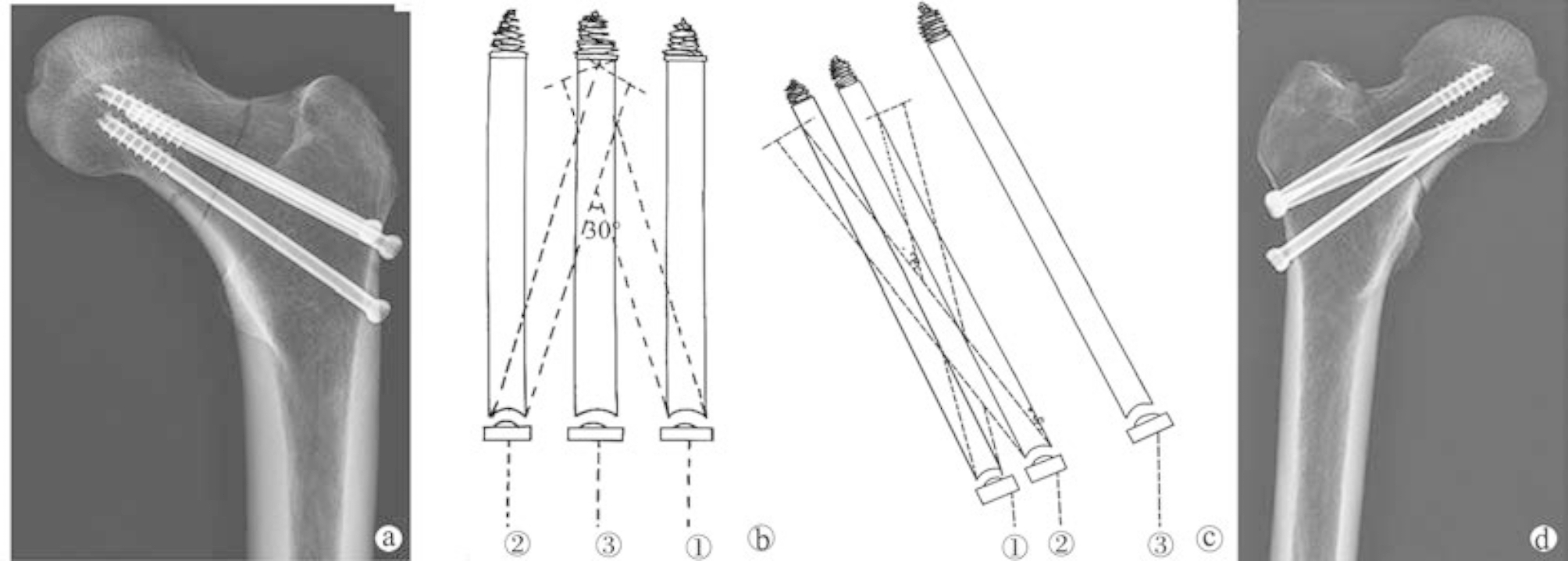
Diagram of inverted triangle and cross-inverted triangle implantation nails a: Inverted triangle implanted nails; b ~ c: Front view and top view of cross-inverted triangle implanted nails. The solid line is the inverted triangle, and the dashed line is the cross-inverted triangle. d: Cross-inverted triangle implanted nails

The nail was placed in an inverted triangular fashion with a pressurized threaded nail. X-ray examination was performed before nailing the left femoral neck fracture specimens in each group to determine the nail position and to analyze the angle between the nail and the femoral stem and the distance between the nailing point and the apex of the greater trochanter. One Kirschner’s pin (No. 3) was drilled parallel to the femoral trochanter 4.00 cm below the trochanter, and two other Kirschner’s pins were drilled 2.50 cm below the trochanter using a parallel guide, with the three pins distributed in an inverted triangle (Fig. 2a). If the placement is ideal, the appropriate length of compression threaded nails is placed parallel to each pin, with the No. 1 and No. 2 compression threaded nails in the same plane. The length of the nail is 0.50-1.00 cm from the femoral head joint surface; if the placement position is not ideal, the nail is redrilled until it is ideal.

The right femoral neck fracture specimen was treated in a left-sided manner, and the same entry point was used to drill the No. 3 Kirschner pin parallel to the femoral trochanter at 4.00 cm below the trochanter. The No. 1 Kirschner pin was drilled 15° upward laterally at 2.50 cm below the trochanter, and the No. 2 Kirschner pin was drilled 15° downward medially so that they were not in the same plane (taking the compression screw nail as an example, Fig. 2b, c). The three needles were distributed in a cross-inverted triangle (Fig. 2d). X-ray fluoroscopy was used to confirm the position of the Kirschner pin. If the position of the Kirschner pin was ideal, a pressurized screw of appropriate length was inserted along each Kirschner pin. If the placement was not ideal, the Kirschner pin was redrilled until ideal.

### Biomechanical index measurement

**Fig. 3:**
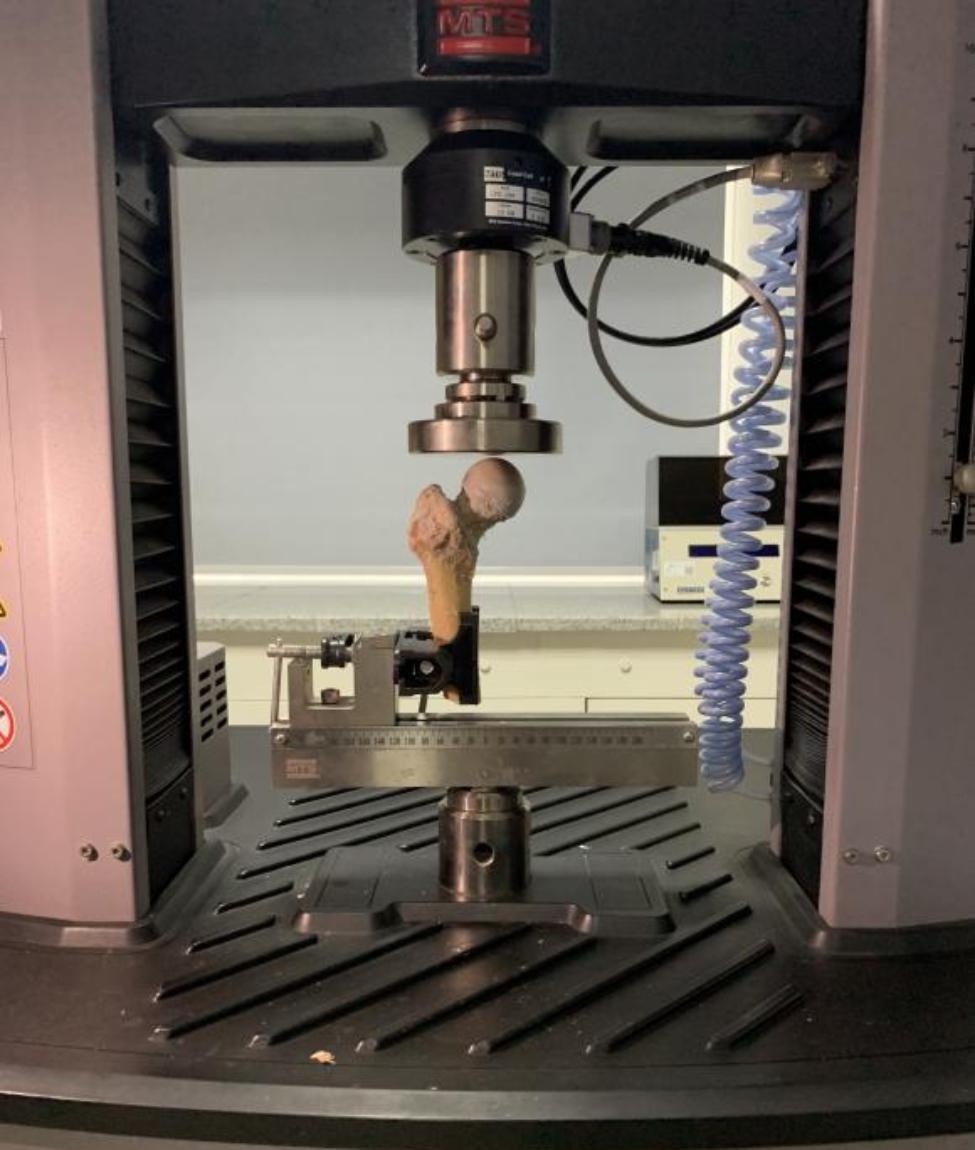
Static pressure test chart of the electronic universal testing machine

The internal fixation model of femoral neck fracture was fixed on the fixture, and the static compression test was performed with an electronic universal testing machine (Fig. 3). The static compression test simulated the force when the femoral neck fracture was weighted on the ground after internal fixation to obtain the axial displacement and maximum load. The pressure-displacement curves were recorded, and it was observed that the load first increased in a fixed proportion with the increase in axial displacement. Then, the curve fluctuated significantly when a critical point was reached, and the load dropped abruptly, which was the maximum load on the femoral neck. The maximum load on the femoral neck and the load at 3.00 mm axial displacement of the femoral head were read according to the curve drawn in the experiment.

### Statistical methods

SPSS 24.0 software was used for statistical analysis. The comparison of the load on the femoral head at an axial displacement of 3.00 mm and the maximum load was performed by the K-S test and LSD-t analysis with a test level of α = 0.05, and P < 0.05 was considered a statistically significant difference.

## Results

**Fig. 4:**
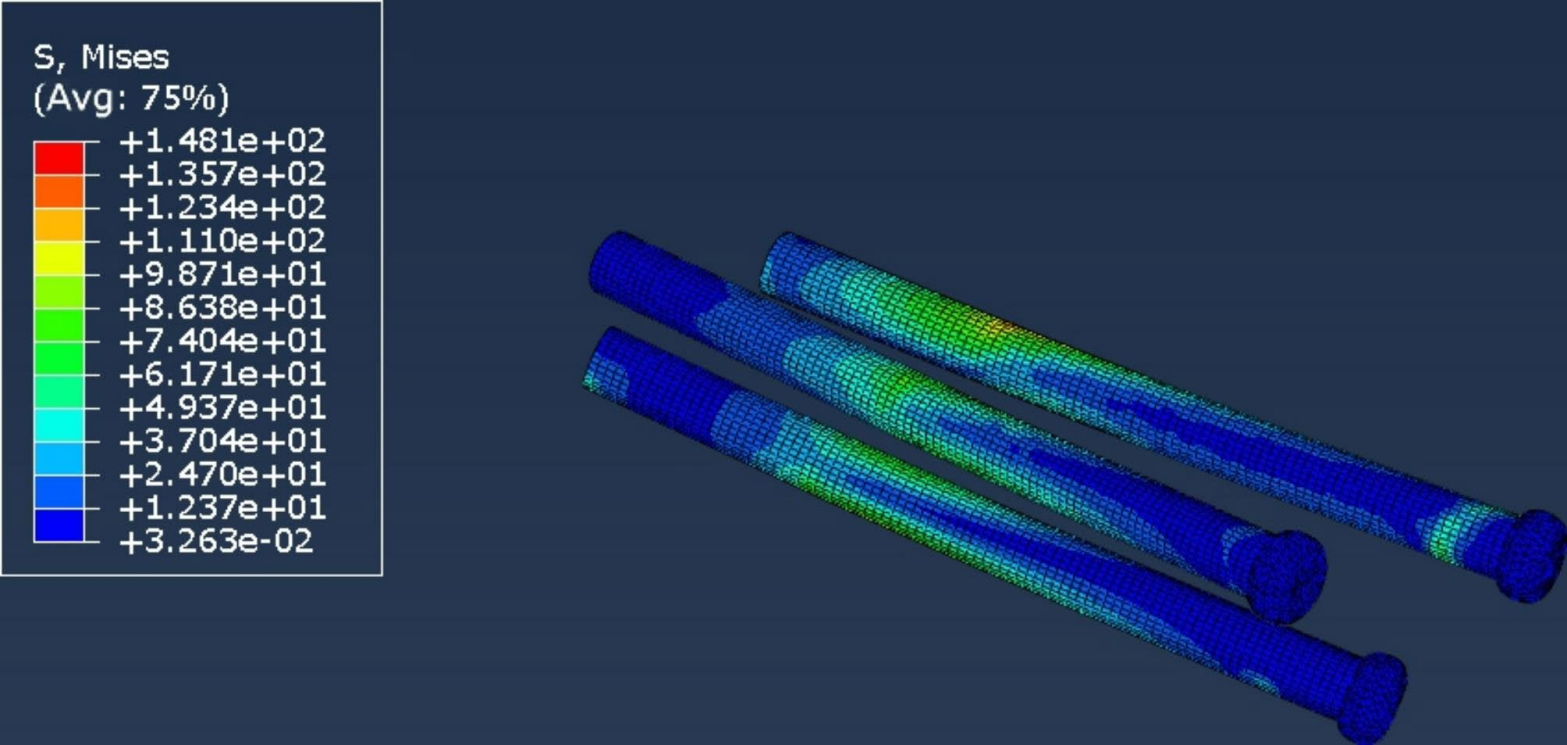
Maximum stress nephogram of the internal fixation device for the cross-inverted triangle internal fixation model

**Fig. 5:**
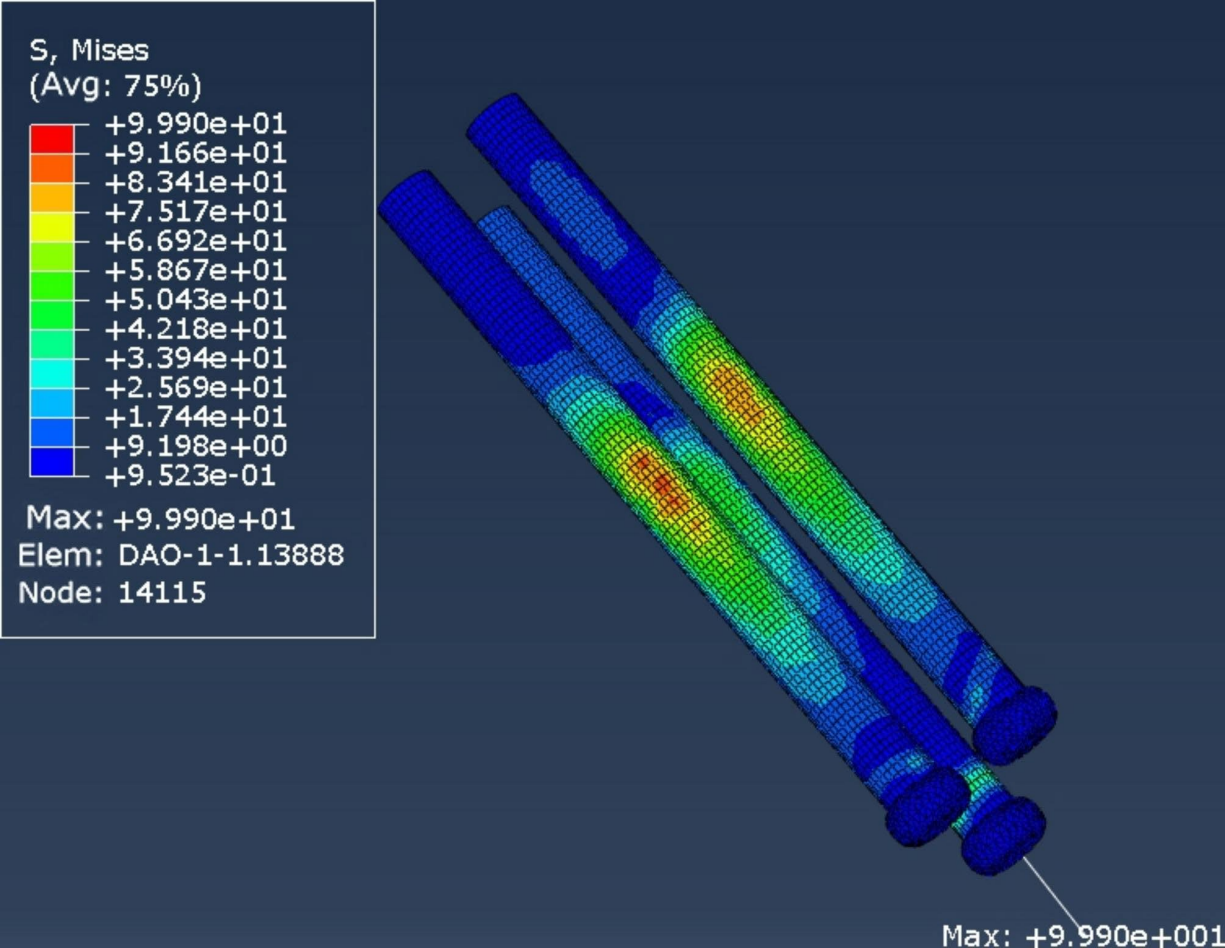
Maximum stress nephogram of the internal fixation device for the inverted triangle internal fixation model

**Fig. 6:**
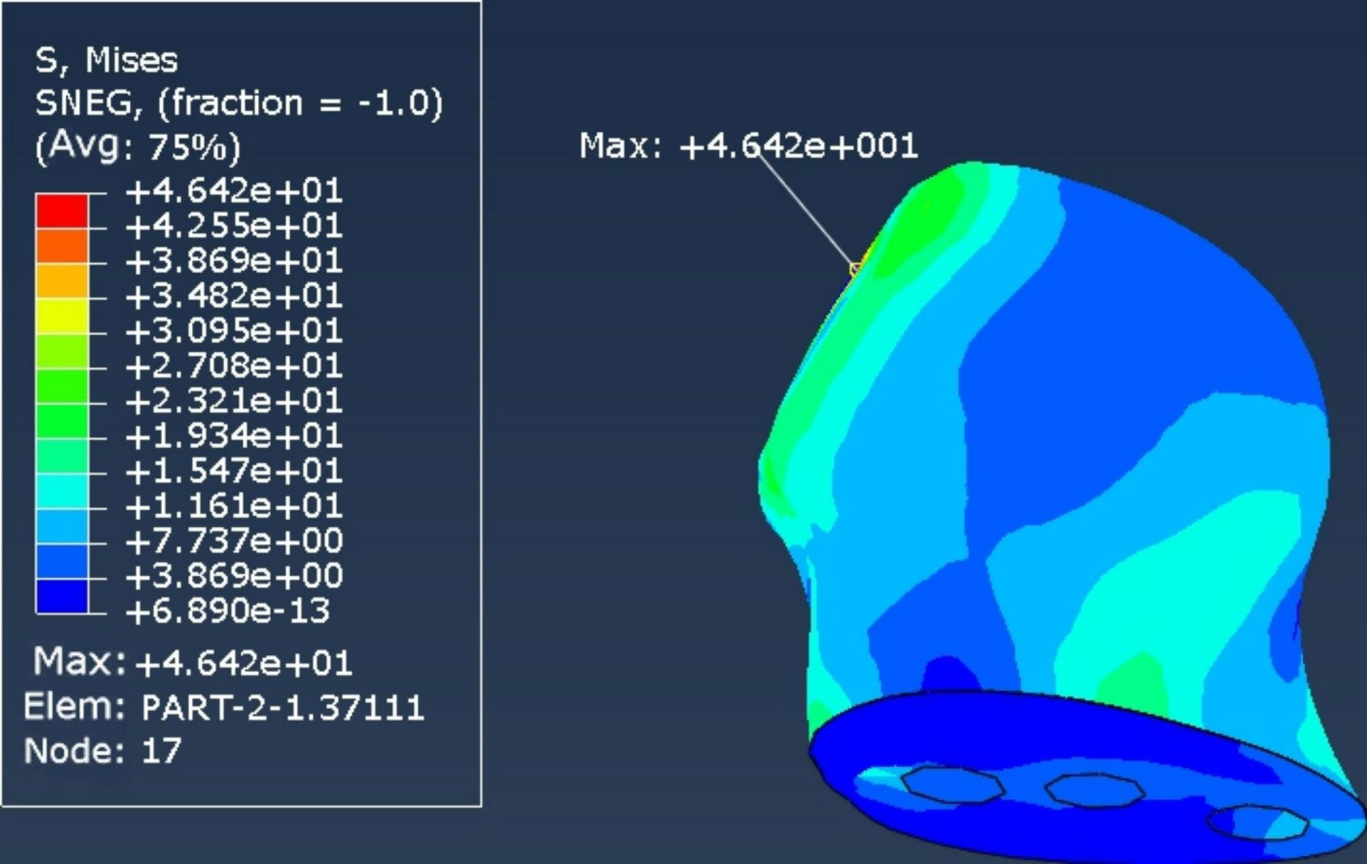
Femoral displacement nephogram of the cross-inverted triangle internal fixation model

**Fig. 7:**
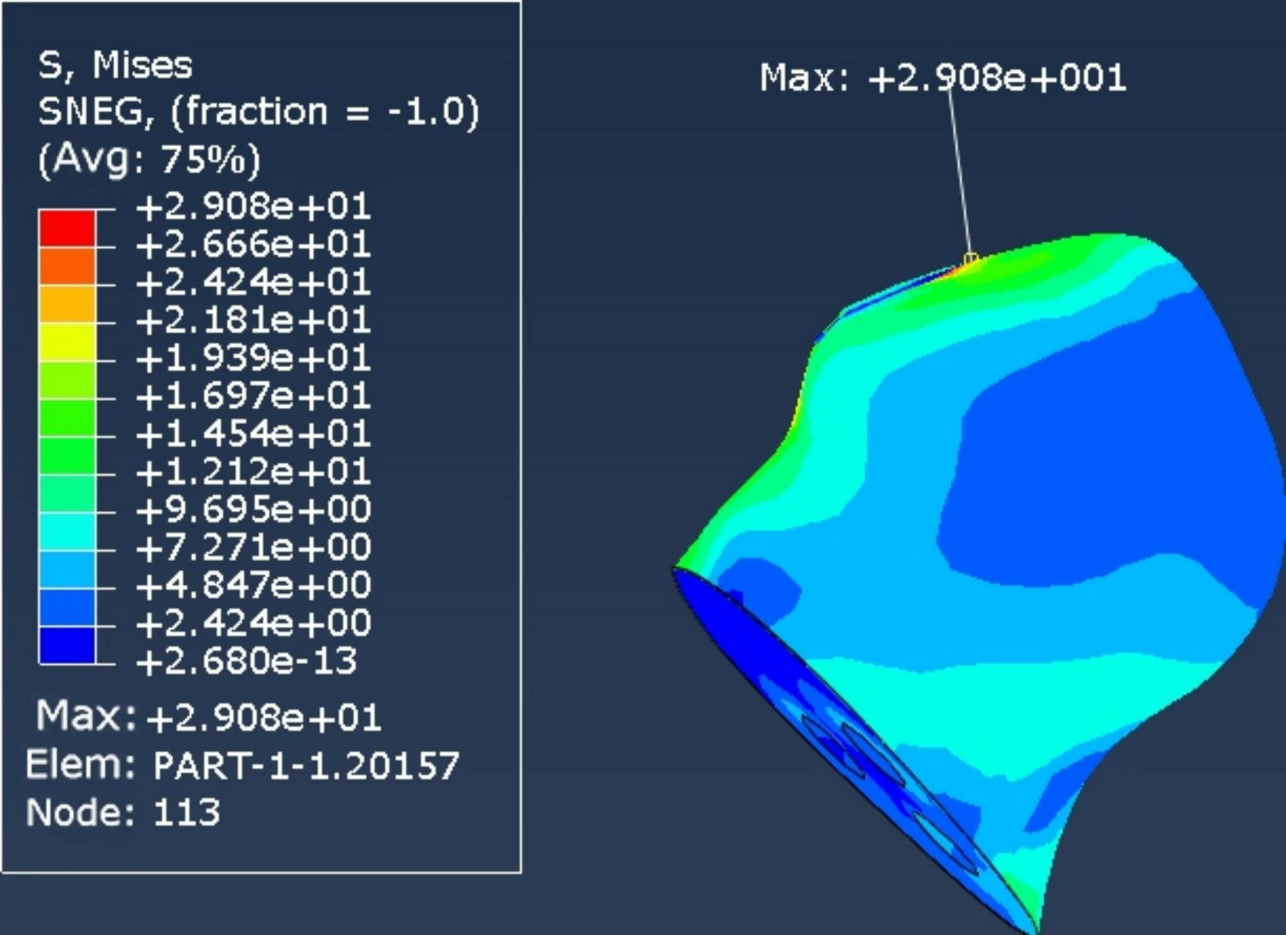
Femoral displacement nephogram of the inverted triangle internal fixation model

The stress peaks of the internal fixation devices of the 2 internal fixation models were mainly concentrated on the screw surfaces near the fracture line. The peak stresses of the internal fixation devices in the cross-inverted triangular hollow screw group and the inverted triangular hollow screw group were 148 and 999 MPa, respectively (Figs. 4 and 5), while the peak femoral displacements in the two internal fixation models were mainly concentrated at the top of the femoral head, with the peak femoral displacements in the cross-inverted triangular hollow screw group and the inverted triangular hollow screw group being 464.2 and 290.8 MPa, respectively (Figs. 6 and 7).

The results of static compression tests showed that the maximum load on the femoral neck and the load at 3.00 mm of axial displacement of the femoral head in the right femur of groups A1, A2, B1, B2 and C2 were greater than those in the left femur, while the maximum load on the femoral neck and the load at 3.00 mm of axial displacement of the femoral head in the right femur of group C1 were less than those in the left femur (Table 1). The mean load at 3.00 mm of axial displacement of the right femoral head was lower than that of the left femoral head. The mean load at 3.00 mm axial displacement of the left femoral head: group C (776.64 N) > group A (710.56 N) > group B (355.86 N), indicating that the compression threaded nail placed in the inverted triangle approach received the highest force in the basal femoral neck fracture, followed by the subcapital type and finally the trans-cranial type. Except for group C1, the cross-inverted triangle approach was superior to the inverted triangle approach.

**Table 1:** Femoral neck fracture specimens with compression threaded nail internal fixation axial compression test results (N)

	A1		A2		B1		B2		C1		C2	
Projects	left	Right	left	Right	left	Right	left	Right	left	Right	left	Right
Load(3.00 mm)	954.45	878.87	1 583.72	542.25	544.39	275.74	635.52	435.98	450.03	681.44	1 011.91	871.83
Maximum load	1525.67	1415.33	1 802.77	901.02	596.38	305.33	1228.89	683.09	602.36	1 023.20	1 471.56	1168.20

The cross-inverted triangle approach was superior to the inverted triangle approach. The differences were not statistically significant (P > 0.05) when comparing the load and the maximum femoral neck load at 3.00 mm of axial displacement of the right femoral head in groups A1 and A2, B1 and B2, and C1 and C2.

The maximum load on the femoral neck and the load on the femoral head at 3.00 mm axial displacement were normally distributed by the K-S test (P = 0.20), but the difference was not statistically significant when the LSD-t test was performed on the load data of the two groups (F = 1.595,=0.235).

## Discussion

In clinical surgery,we always classified femoral neck fractures into subcapital, basal and trans-cranial types according to the anatomical location of the fracture. In the AO classification of femoral neck fracture, type B1 was subcapital fracture with no or slight displacement. B2 was a trans-cervical fracture, and the fracture line was located in the middle or base of the femoral neck. B3 is a subcephalic fracture without compression.This type of fracture is usually seen in elderly patients and has a high probability of ischemic necrosis of the femoral head and a poor [14]; in the trans-neck type, the entire fracture surface passes through the femoral neck, which is less common; in the basal type, the fracture line is located at the base of the femoral neck, and the fracture end has good blood flow, which easily maintains stability after repositioning, and the fracture heals easily and has a good prognosis. Currently, patients are usually treated with three pressurized threaded nails placed in an inverted triangular fashion. However, [15] showed that the biomechanical performance of the 3 hollow compression screws was poor in fixing high vertical shear stress Pauwels type III femoral neck fractures, which resulted in a high failure rate and indicated that there are still some shortcomings in this method of nailing.

The stability of internal fixation directly affects the occurrence of femoral neck shortening and fracture healing, and the layout of screws is closely related to the biomechanical stability of the fixed [16, 17]. To make the internal fixation more effective, the first consideration is the area of fixation under the same condition of internal fixation; the larger the area of fixation, the larger the area of force bearing, and the larger the pressure that can be withstood. Therefore, the research team designed a new solution of cross-inverted triangle placement, in which the two pressurized threaded nails above the inverted triangle method are placed in a crossed manner, forming two triangular planes.

The experimental results of the finite element analysis showed that the cross-inverted triangular hollow threaded nail internal fixation device was subjected to lower maximum stress and higher peak femoral displacement, and the stress distribution in the structure of the internal fixation system was more uniform and distributed than that of the inverted triangular hollow threaded nail model, which indicated that the cross-inverted triangular hollow threaded nail had better conductivity and more stable fixation than the inverted triangular hollow threaded nail. Therefore, the cross-inverted triangular hollow thread nail can effectively reduce the risk of postoperative fracture end displacement and internal fixation failure, and our internal fixation method is theoretically superior.

The biomechanical analysis showed that the mean load at 3.00 mm of axial displacement of the femoral head was highest in the inverted triangle placement of the compression threaded nail, followed by the subcranial type and finally the trans-cervical type, which was slightly different from other studies, probably due to the small sample size. In the cross-inverted triangle placement of the pressurized threaded nail, the force of the subcranial and trans-cervical types was better than that of the inverted triangle, while the basal type was inferior to the inverted triangle, which may be caused by the poor bone quality of the C1 group. The experimental data of this study showed that the stability of the crossed inverted triangle approach was better than that of the inverted triangle approach when used to fix subcapital and transcervical fractures, while the stability of the crossed inverted triangle approach was inferior to that of the inverted triangle approach when used to fix basal fractures, and there was no significant difference between men and women in the application of the crossed inverted triangle approach.

The results of this study showed that the mean load at 3.00 mm of axial displacement of the femoral head was highest in the inverted triangle placement of the compression threaded nail for the basal type, followed by the inferior cephalic type, and finally the transcervical type, which was slightly different from the results of other studies, probably due to the small sample [18]. In the cross-inverted triangle placement of the pressurized threaded nail, the force of the subcranial and transcervical types was better than that of the inverted triangle, while the basal type was inferior to the inverted triangle, which may be caused by the poor bone quality of the C1 group.

## Conclusion

The experimental data of this study showed that the stability of the crossed inverted triangle approach was better than that of the inverted triangle approach when used for fixing subcranial and transcervical fractures, and the stability of the crossed inverted triangle approach was inferior to that of the inverted triangle approach when used for fixing basal fractures.

## Limitations

In this study, the sample size of the femur was small, and the specimen was placed for a long time. However, all femurs in this experiment were under the same conditions, the final data analysis method was a cross-sectional control within the group, and there was no significant difference in the natural factor conditions of the femurs in the same group, which can have a good control effect. In contrast, the femur in group C1 may have been poorly preserved and poorly fixed before the experiment, resulting in deviations from the expected results. We will further expand the number of femur specimens for further experimental verification.

## Data Availability

All data generated or analysed during this study are included in this published article [and its supplementary information files].
